# Supplementary material for: A popular Indian clove-based mosquito repellent is less effective against Culex quinquefasciatus and Aedes aegypti than DEET
Source: PLoS One. 2019 Nov 5;14(11):e0224810. doi: 10.1371/journal.pone.0224810 (PMC6830767; doi:10.1371/journal.pone.0224810)
Supplement: S1 Dataset — This file contains all raw data from chemical analysis and behavioral measurements that were used to generated Figs 3 and 4, respectively. (PDF) [file pone.0224810.s001.pdf]

**Raw Data for Figure 3**		
**Portuense**		
Day-4	Day-5	Day-6
6.41	7.9	11.68
4.49	7.01	9.48
4.23	5.71	7.06
**Beija Flor**		
Day-4	Day-5	Day-6
11.71	15.27	15.33
12.78	15.56	19.1
10.45	13.41	15.92
**Kitano**		
Day-4	Day-5	Day-6
11.71	15.27	15.33
12.78	15.56	19.1
10.45	13.41	15.92

Raw Data for Figure 4				
**Panel A- *Cx. quinquefasciatus***				
1% DEET	Solvent		Extract (BF)	Solvent
4	22		6	28
1	26		9	30
2	22		7	20
3	27		6	26
1	15		5	18
2	17		6	20
3	11		0	4
0	10		7	11
**Panel B- *Cx. quinquefasciatus***				
1% DEET	Solvent		1% Eugenol	Solvent
0	12		1	9
0	11		3	6
0	7		3	10
0	7		2	9
0	10		3	9
0	9		4	9
1	9		0	6
0	7		0	9
**Panel C - *Ae. aegypti***				
1% DEET	Solvent		Extract (BF)	Solvent
6	28		7	28
8	20		10	23
5	16		9	21
2	18		10	18
2	12		4	8
4	17		6	13
3	18		6	21
5	19		8	12
2	14		6	17
4	13		4	15
